# A Nonpediatric Extraosseous Ewing Sarcoma of the Pancreas: Differential Diagnosis and Therapeutic Strategies

**DOI:** 10.1155/2020/2792750

**Published:** 2020-01-30

**Authors:** Binoy Yohannan, Mark Feldman

**Affiliations:** Department of Internal Medicine, Texas Health Presbyterian Hospital Dallas, Texas 75231, USA

## Abstract

Extraosseous Ewing's sarcoma is a rare and aggressive malignant tumor with a poor prognosis. The pancreas is an extremely uncommon primary site, with only 27 cases that have been published worldwide. We report a 26-year-old female who presented with 5 days of left upper quadrant pain, nausea, and vomiting. On examination, she was anicteric and had epigastric and left upper quadrant tenderness without guarding, rebound tenderness, or a palpable mass. She had slightly elevated serum aminotransferase and lipase levels. Abdominal computerized tomography revealed a multilobulated tumor arising from the body and tail of the pancreas. A biopsy confirmed a small round cell tumor, and immunohistochemistry was positive for CD99 in approximately 70% of the tumor cells. A fluorescence in situ hybridization (FISH) assay showed a 22q12 rearrangement. She was diagnosed with extraosseous Ewing sarcoma of the pancreas and underwent multiagent neoadjuvant chemotherapy followed by surgical resection, but subsequent imaging revealed evidence of systemic disease progression. She chose to go on hospice care and died a few weeks later.

## 1. Introduction

The Ewing sarcoma (ES) family of tumors (ESFT) includes ES of bone (ESB) and extraosseous ES (EES) [1]. ESB was first described by James Ewing in 1921 [2]. EES was first reported by Tefft et al. in 1969 [3]. ESB is more commonly seen in males, with a peak age between 10 and 20. EES is also most commonly seen in the second decade of life, but all age groups can be affected, and there is no gender predilection [4].

Approximately 30% of all ESFTs are extraosseous, most commonly arising in the soft tissues of the trunk or extremities, but rarely in various locations in the gastrointestinal tract including the biliary tree, stomach, esophagus, and oral cavity. The pancreas is an extremely uncommon extraosseous location, with only 27 cases reported worldwide [5, 6]. We report a lethal case of cytogenetically confirmed EES of the pancreas in a young woman who presented with abdominal pain.

## 2. Case Presentation

**Figure 1:**
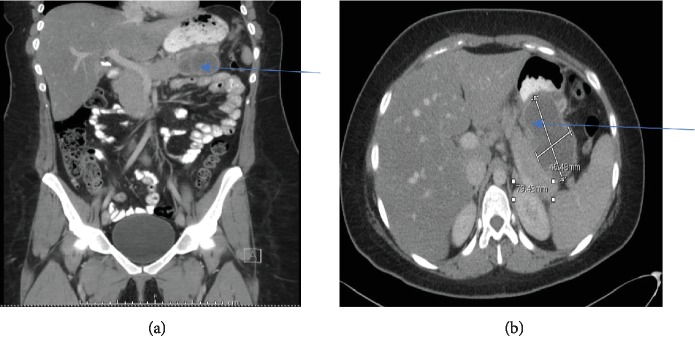
CT of the abdomen and pelvis with oral and IV contrast showing a large multilobulated upper abdominal mass (arrow) inseparable from the body and tail of the pancreas (a). The tumor (10 cm × 9 cm × 7 cm) filled the lesser sac (arrow) and wrapped around the gastric fundus (b).

**Figure 2:**
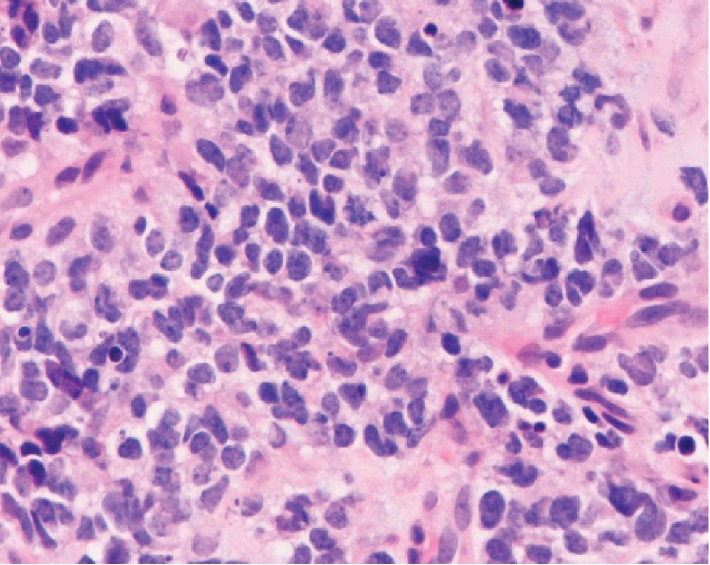
Histopathology with hematoxylin and eosin staining showing small round blue cells with hyperchromatic nuclei and scant amounts of ill-defined cytoplasm (×100).

A 26-year-old Caucasian woman presented with 5 days of left upper quadrant abdominal pain, nausea, and vomiting. She had no fever, chills, night sweats, jaundice, dysphagia, early satiety, constipation, melena, hematochezia, anorexia, or weight loss. She denied tobacco or alcohol use, radiation exposure, or family history of cancer. Her vital signs were normal. She had no pallor, icterus, or lymphadenopathy. Tenderness was present in the epigastrium and left upper quadrant without guarding, rebound tenderness, or palpable mass. She had normal bowel sounds. Rectal and pelvic examinations were negative. She had a normal complete blood count, coagulation profile, and inflammatory markers. Serum testing revealed slightly elevated AST, ALT, and lipase levels. Computerized tomography of the abdomen with oral and intravenous contrast showed a large (10 cm × 9 cm × 7 cm) multilobulated upper abdominal mass inseparable from the body and tail of the pancreas, filling the lesser sac, and wrapping around the gastric fundus (Figure 1). Positron emission tomography-CT scan confirmed a fludeoxyglucose F 18-avid pancreatic mass and showed a separate 2.2 cm left subdiaphragmatic nodule suspicious for metastatic disease. She underwent a CT-guided core biopsy of the pancreatic mass which showed small round cells with hyperchromatic nuclei and scant amounts of ill-defined cytoplasm (Figure 2). Immunohistochemistry was positive for CD99 (membrane staining pattern in approximately 70% of tumor cells), cytokeratin AE1/AE3, NKX2.2, CD 56, SOX10, and synaptophysin. A FISH assay of tumoral tissue showed a 22q12 rearrangement. A diagnosis of extraosseous ES of the pancreas was made. The patient then received 5 cycles of neoadjuvant chemotherapy with vincristine, ifosfamide, and doxorubicin. However, a postchemotherapy CT showed evidence of disease progression. She then underwent laparotomy which showed a dominant retrogastric tumor mass involving 70 percent of the posterior gastric wall, a 3.5 cm separate lesion just left of the pancreatic anatomic neck, and two soft tissue pericolic lesions adjacent to the splenic flexure. An en bloc distal pancreatectomy with splenectomy, subtotal gastrectomy with Roux-en-Y gastrojejunostomy, and left colectomy with primary anastomosis were performed. Surgical histopathology demonstrated residual ES with only 30% tumor necrosis and a continued high mitotic rate (>20 mitoses/10 HPF). Resected margins were negative for a tumor. Postoperative CT showed evidence of disease recurrences in the right diaphragmatic crus and the rectosigmoid junction. She received 3 additional cycles of adjuvant chemotherapy with vincristine, temozolomide, and irinotecan, but subsequent CT imaging showed metastatic disease in the liver and retroperitoneum. She chose hospice care and died a few weeks later.

## 3. Discussion

EES of the pancreas is a rare and aggressive malignant tumor with a poor prognosis. They usually arise from the head of the pancreas [5]. The most common sign or symptom is abdominal pain, followed in frequency by jaundice [5]. Affected patients can present with anemia, or rarely, hyperglycemia or precocious puberty [5]. When compared to the infiltrative growth pattern of pancreatic adenocarcinoma, EES of the pancreas has an expansile growth pattern; it tends to present late in the disease course [6]. Histologically, there are small round cells (blue on H&E stain) with hyperchromatic nuclei and scant cytoplasm containing neuronal secretory granules, neurofilaments, and pyknotic nuclear granules [7]. These malignant cells show a high expression of single-chain type-1 glycoprotein (MIC2, also called CD99) and are one of the best diagnostic immunohistochemical markers for this disease [8]. Other immunohistochemical markers include O13, HBA71, 12E7, RFB1, and neuronal markers such as neuron specific enolase, chromogranin A, and synaptophysin. In 90% of cases, these tumors share a unique and specific 11;22 chromosomal translocation that involves fusion between *EWS* on chromosome 22 and *FLI-1* on chromosome 11. This chimeric gene product can be detected by FISH or reverse transcription polymerase chain reaction. In the rest of the cases, there is a mutation or rearrangement in *ERG* on chromosome 22 [7, 9].

The differential diagnosis of a small round blue cell tumor includes Wilms' tumor, neuroblastoma, hepatoblastoma, rhabdomyosarcoma, small-cell lymphoma, visceral small-cell neuroendocrine carcinoma, desmoplastic small round cell tumor, pancreatic neuroendocrine tumor, and pancreatoblastoma, with the latter four arising from the pancreas [10].

On ultrasonography, EES lesions are most frequently hypoechoic, although anechoic areas may also be present, likely representing areas of hemorrhage or necrosis [11]. EES appears hypodense or isodense on noncontrast CT depending on the degree of necrosis; one-third of tumors may have calcification. On MRI, these tumors are isointense on T1-weighted images and either isointense or hyperintense on T2-weighted images. The tumors have irregular shapes with ill-defined borders and have heterogeneous enhancement with contrast [12]. Bone metastasis can be better detected by FDG-PET rather than by bone scintigraphy, whereas contrast-enhanced helical CT is superior to FDG-PET for the detection of pulmonary metastases due to the small size of the pulmonary nodules [13].

The diagnosis of EES is based on clinical symptoms, histopathology, immunohistochemical features, and cytogenetic analysis. A core biopsy of the tumor is usually sufficient for diagnostic purposes; however, immunohistochemistry and cytogenetic analysis are essential to rule out other small round cell tumors listed earlier.

EES is a highly aggressive tumor, and almost all patients have occult or gross disseminated disease at the time of diagnosis. Prior to the era of chemotherapy, the prognosis was dismal, but survival has improved substantially with the advent of chemotherapy. It is crucial that affected patients are treated with a multidisciplinary approach at centers of excellence with expertise in managing these rare tumor types. The standard treatment is systemic neoadjuvant or adjuvant multiagent chemotherapy combined with surgery and radiotherapy [4, 14]. With ES being a rare tumor in adults, most chemotherapy protocols have been adapted from published pediatric studies. Unfortunately, adult patients with ES do poorly when compared to children treated with the same regimen. The popular chemotherapy regimen includes vincristine, doxorubicin (or dactinomycin), and cyclophosphamide (VDC) [15]. Adding alternate cycles of ifosfamide and etoposide (IE) to the standard regimen significantly improves the outcome for patients with localized disease but does not affect the outcome for patients with metastatic disease [16]. Alternately, a VDI regimen (vincristine, doxorubicin, and ifosfamide) in the neoadjuvant setting, as used in our patient, followed by an adjuvant therapy based on posttreatment percent necrosis has a favorable outcome when compared to historical adult controls treated with VDC-IE [17]. Local control of disease can be achieved surgically with negative tumor margins or with radiation therapy in unresectable tumors or patients with positive tumor margins. Aggressive surgical treatment with negative surgical margins is associated with overall survival benefit [18]. The dose of radiation recommended for EES is 45 Gy to the initial tumor volume plus a 2 cm margin area, followed by a boost of 5.4 Gy to a total dose of 50.4 Gy [15].

Despite these aggressive measures, the outcome of EES is unsatisfactory with an overall 5-year survival rate of 60-80% in patients with localized disease and below 30% in patients with metastatic disease at the time of diagnosis [19]. Also, the existing chemotherapies are highly toxic and poorly tolerated by patients. Hence, there is an unmet need for newer therapies with better clinical response and a favorable safety profile.

Pazopanib, a multitargeted tyrosine kinase inhibitor, is approved for the treatment of advanced soft tissue sarcomas, including EES [20]. There are few case reports of successful treatment of retroperitoneal and paravertebral EES with pazopanib [21, 22]. Assuming that this data can be extrapolated to EES of the pancreas, pazopanib could provide a new treatment option for patients who have failed multiple lines of therapy. Furthermore, there are newer promising drugs with a novel mechanism of action that could potentially transform the treatment paradigm for ES in the future. The *EWS-FLI1* is a tumor-specific translocation seen in ESFT and has a great potential as a molecular target for therapy. However, *EWS-FLI1* has been a very difficult target to analyze *in vitro* due to poor solubility [23]. TK216 is a novel small-molecule that directly binds to *EWS-FLI1* and inhibits its function by blocking binding to RNA helicase A. TK216 demonstrates potent antiproliferative effects on ES cell lines and xenografts and is currently being tested in clinical trials [24]. Another exciting target is the enzyme lysine-specific demethylase 1 (LSD1) which is highly expressed in ES cell lines, and inhibitors of LSD1 could offer a ray of hope against this lethal disease [25]. In preclinical studies, it has been shown that focal adhesion kinase (FAK) inhibitors and Aurora kinase B inhibitors synergistically impair Ewing sarcoma cell growth and significantly inhibit tumor progression; this treatment approach needs to be validated in clinical trials [26]. Intriguingly, *in vitro* studies have shown that ES cells are highly sensitive to cyclin-dependent kinase (CDK) 7/12/13 inhibitors that impair DNA damage repair in fusion-positive ES [27]. Also, CDK12/13 inhibitors and PARP inhibitors such as olaparib are highly synergistic in ES [27]. Ganitumab (monoclonal antibody against insulin growth factor-1R) has been granted an orphan drug designation by the FDA and is currently being investigated in phase III clinical trials as a first-line therapy for patients with newly diagnosed metastatic ES (http://www.fda.gov). The other novel drugs that are being studied include a NEDD8-activating enzyme (NAE) inhibitor (pevonedistat), immune checkpoint inhibitors, and chimeric antigen receptor (CAR) T-cell therapy.

## 4. Conclusion

Extraosseous Ewing's sarcoma is a rare soft tissue sarcoma with poor prognosis. This case highlights the importance of considering EES in the differential diagnosis of a young patient presenting with a pancreatic mass. Despite aggressive therapy, the outcome remains unsatisfactory and there is an unmet need for newer therapies.

## References

[1] Carvajal R., Meyers P. (2005). Ewing's Sarcoma and Primitive Neuroectodermal Family of Tumors. *Hematology/oncology Clinics of North America*.

[2] Ewing J. Diffuse endothelioma of bone.

[3] Tefft M., Vawter G. F., Mitus A. (1969). Paravertebral ‘round cell’ tumors in children. *Radiology*.

[4] Ahmad R., Mayol B. R., Davis M., Rougraff B. T. (1999). Extraskeletal Ewing’s sarcoma. *Cancer*.

[5] Bose P., Murugan P., Gillies E., Holter J. L. (2012). Extraosseous Ewing's sarcoma of the pancreas. *International Journal of Clinical Oncology*.

[6] Nishizawa N., Kumamoto Y., Igarashi K. (2015). A peripheral primitive neuroectodermal tumor originating from the pancreas: a case report and review of the literature. *Surgical Case Reports*.

[7] Grünewald T. G. P., Cidre-Aranaz F., Surdez D. (2018). Ewing sarcoma. *Nature Reviews Disease Primers*.

[8] Rocchi A., Manara M. C., Sciandra M. (2010). CD99 inhibits neural differentiation of human Ewing sarcoma cells and thereby contributes to oncogenesis. *The Journal of Clinical Investigation*.

[9] Yamaguchi U., Hasegawa T., Morimoto Y. (2005). A practical approach to the clinical diagnosis of Ewing’s sarcoma/primitive neuroectodermal tumour and other small round cell tumours sharing EWS rearrangement using new fluorescence in situ hybridisation probes for EWSR1 on formalin fixed, paraffin wax embedded tissue. *Journal of Clinical Pathology*.

[10] Chen Q. R., Vansant G., Oades K. (2007). Diagnosis of the small round blue cell tumors using multiplex polymerase chain reaction. *The Journal of Molecular Diagnostics*.

[11] O'Keeffe F., Lorigan J. G., Wallace S. (1990). Radiological features of extraskeletal Ewing sarcoma. *The British Journal of Radiology*.

[12] Robbin M. R., Murphey M. D., Jelinek J. S., Temple H. T. (1998). Imaging of soft tissue Ewing sarcoma and primitive neuroectodermal tumor. *Radiology*.

[13] Györke T., Zajic T., Lange A. (2006). Impact of FDG PET for staging of Ewing sarcomas and primitive neuroectodermal tumours. *Nuclear Medicine Communications*.

[14] Rud N. P., Reiman H. M., Pritchard D. J., Frassica F. J., Smithson W. A. (1989). Extraosseous Ewing’s sarcoma. A study of 42 cases. *Cancer*.

[15] Granowetter L., Womer R., Devidas M. (2009). Dose-intensified compared with standard chemotherapy for nonmetastatic Ewing sarcoma family of tumors: a Children's Oncology Group Study. *Journal of Clinical Oncology*.

[16] Grier H. E., Krailo M. D., Tarbell N. J. (2003). Addition of ifosfamide and etoposide to standard chemotherapy for Ewing's sarcoma and primitive neuroectodermal tumor of bone. *The New England Journal of Medicine*.

[17] Wagner M. J., Gopalakrishnan V., Ravi V. (2017). Vincristine, ifosfamide, and doxorubicin for initial treatment of Ewing sarcoma in adults. *The Oncologist*.

[18] Raney R. B., Asmar L., Newton W. A. (1997). Ewing's sarcoma of soft tissues in childhood: a report from the Intergroup Rhabdomyosarcoma Study, 1972 to 1991. *Journal of Clinical Oncology*.

[19] Eralp Y., Bavbek S., Başaran M. (2002). Prognostic factors and survival in late adolescent and adult patients with small round cell tumors. *American Journal of Clinical Oncology*.

[20] van der Graaf W. T. A., Blay J.-Y., Chawla S. P. (2012). Pazopanib for metastatic soft-tissue sarcoma (PALETTE): a randomised, double-blind, placebo-controlled phase 3 trial. *The Lancet*.

[21] Yamamoto Y., Nozawa M., Shimizu N., Minami T., Yoshimura K., Uemura H. (2014). Pazopanib for recurrent extraosseous Ewing's sarcoma of the retroperitoneum. *International Journal of Urology*.

[22] Alcindor T. (2015). Response of refractory Ewing sarcoma to pazopanib. *Acta Oncologica*.

[23] Üren A., Toretsky J. A. (2005). Ewing’s sarcoma oncoprotein EWS–FLI1: the perfect target without a therapeutic agent. *Future Oncology*.

[24] Federman N., Meyers P. A., Daw N. C. (2017). A phase I, first-in-human, dose escalation study of intravenous TK216 in patients with relapsed or refractory Ewing sarcoma. *Journal of Clinical Oncology*.

[25] Pishas K. I., Drenberg C. D., Taslim C. (2018). Therapeutic targeting of KDM1A/LSD1 in Ewing sarcoma with SP-2509 engages the endoplasmic reticulum stress response. *Molecular Cancer Therapeutics*.

[26] Wang S., Hwang E. E., Guha R. (2019). High-throughput chemical screening identifies focal adhesion kinase and Aurora kinase B inhibition as a synergistic treatment combination in Ewing sarcoma. *Clinical Cancer Research*.

[27] Iniguez A. B., Stolte B., Wang E. J. (2018). EWS/FLI confers tumor cell synthetic lethality to CDK12 inhibition in Ewing sarcoma. *Cancer Cell*.

